# Selective Neuronal and Brain Regional Expession of IL-2 in IL2P 8-GFP Transgenic Mice: Relation to Sensorimotor Gating

**DOI:** 10.4172/2161-0460.1000127

**Published:** 2013-10-28

**Authors:** Danielle Meola, Zhi Huang, John M Petitto

**Affiliations:** Departments of Psychiatry, Neuroscience, and Pharmacology & Therapeutics, McKnight Brain Institute, USA

**Keywords:** Interleukin-2, Congenic mice, Sensorimotor gating, Prepulse inhibition

## Abstract

Brain-derived interleukin-2 (IL-2) has been implicated in diseases processes that arise during CNS development (e.g., autism) to neurodegenerative alterations involving neuroinflammation (e.g., Alzheimer’s disease). Progress has been limited, however, because the vast majority of current knowledge of IL-2’s actions on brain function and behavior is based on the use exogenously administered IL-2 to make inferences about the function of the endogenous cytokine. Thus, to identify the cell-type(s) and regional circuitry that express brain-derived IL-2, we used B6.Cg-Tg/ IL2-EGFP17Evr (IL2p8-GFP) transgenic mice, which express green fluorescent protein (GFP) in peripheral immune cells known to produce IL-2. We found that the IL2-GFP transgene was localized almost exclusively to NeuN-positive cells, indicating that the IL-2 is produced primarily by neurons. The IL2-GFP transgene was expressed in discrete nuclei throughout the rostral-caudal extent of the brain and brainstem, with the highest levels found in the cingulate, dorsal endopiriform nucleus, lateral septum, nucleus of the solitary tract, magnocellular/gigantocellular reticular formation, red nucleus, entorhinal cortex, mammilary bodies, cerebellar fastigial nucleus, and posterior interposed nucleus. Having identified IL-2 gene expression in brain regions associated with the regulation of sensorimotor gating (e.g., lateral septum, dorsal endopiriform nucleus, entorhinal cortex, striatum), we compared prepulse inhibition (PPI) of the acoustic startle response in congenic mice bred in our lab that have selective loss of the IL-2 gene in the brain versus the peripheral immune system, to test the hypothesis that brain-derived IL-2 plays a role in modulating PPI. We found that congenic mice devoid of IL-2 gene expression in both the brain and the peripheral immune system, exhibited a modest alteration of PPI. These finding suggest that IL2p8-GFP transgenic mice may be a useful tool to elucidate further the role of brain-derived IL-2 in normal CNS function and disease.

## Introduction

Research has implicated both peripheral immune and brain-derived interleukin-2 (IL-2) in neurologic disease processes that arise during CNS development (e.g., autism) to neurodegenerative alterations involving neuroinflammation (e.g., Alzheimer’s disease) [1]. It is widely appreciated, for example, that exogenously administered IL-2 has neuromodulatory actions ranging from neurotrophic effects on septohippocampal neurons in culture [2,3] and neurotransmitter release from cholinergic neurons [4,5], to hippocampal long-term potentiation [6] and age-related changes in learning and memory [7]. The vast majority of our knowledge of IL-2’s actions on brain function and behavior, however, is based on studies that use exogenously administered IL-2 to make inferences about the function of the endogenous cytokine.

Despite interest for many years about IL-2 as a neurotrophic factor and neuromodulator, reliable tools have been lacking to identify the cellular origin of brain-derived IL-2 and the circuitry involved in endogenous IL-2’s actions. IL-2-like immunoreactivity has been mapped to discrete areas of the normal rat forebrain [8], and IL-2 has been measured in rat and human hippocampal tissue extracts using radioimmunoassay [9,10]. Unfortunately, the techniques used in those early studies (e.g. antiserum directed against recombinant human IL-2) resulted in poor resolution and IL-2 reactivity appeared nonspecific, and did not coincide with the distribution of IL-2 receptors identified later by in situ hybridization [11,12]. As UA-rich IL-2 mRNA transcripts in mice are unstable, their identification *in vivo* has been challenging. Although IL-2 mRNA has been detected in the striatum and prefrontal cortex of rats [13,14], use of in situ hybridization to identify cell-specific and regional IL-2 gene expression in mice by our lab and others has been unsuccessful. To date, the literature has been inconclusive and yielded conflicting or nonspecific results. At present, there is no reliable method to track IL-2 mRNA expression in situ in the brain to elucidate further the role of brain-derived IL-2 in CNS function and disease.

B6.Cg-Tg/IL2-EGFP17Evr (IL2p8-GFP) transgenic mice, generated by targeting a new upstream regulatory region of the IL-2 gene, reliably express green fluorescent protein (GFP) in immune cells known to produce IL-2 [15]. Since it has not been possible to reliably identify the cellular source and regional gene expression of the brain IL-2 gene using conventional in situ hybridization histochemistry in mice, we conducted this study to address these issues. The expression of GFP in the brains of these transgenic animals has not been documented. Here we report on the expression of GFP from the brains of IL2p8-GFP transgenic mice, a potentially powerful tool to reliably assess the cellular source and location of IL-2 gene expression in the mouse brain. Since GFP has a significantly longer half-life than IL-2, we postulated that these transgenic mice should provide a clear account of endogenous brain IL-2 expression without the problems encountered with other methods (e.g., problems regarding limits of detection, cross-reactivity with other cytokines, high levels of nonspecific background staining). In the present study, we first performed fluorescent immunohistochemistry co-labeling techniques to determine which brain cell types (i.e. neurons or glia), and which brain regions throughout the rostral-caudal extent of the brain and brainstem, express the IL2-GFP transgene. We then sought to use this information to determine if the expression pattern of IL-2 is linked to behavioral functions known to be associated with the underlying neural circuitry [16]. In this study, having identified IL-2 gene expression in brain regions associated with regulation of sensorimotor gating (e.g., lateral septum, dorsal endopiriform nucleus, entorhinal cortex, striatum), we then compared prepulse inhibition (PPI) of the acoustic startle response (a measure of sensorimotor gating) in congenic mice with selective loss of the IL-2 gene in the brain versus the peripheral immune system to test the hypothesis that brain-derived IL-2 plays a role in modulating PPI. To accomplish this goal, we used a strategy described previously by our lab where we bred congenic mice on the severe combined immunodeficient (SCID) background. SCID mice have defective peripheral T and B cells and cannot produce peripheral IL-2 [17]. Thus, we bred congenic strains of C57BL/*6scid*-IL-2−/− (IL2-KO/SCID) and C57BL/*6scid*-IL-2+/+ (WT/ SCID) mice; the former do not produce IL-2 in either the brain or peripherally, and the latter produce IL-2 in the brain exclusively. Since IL-2 gene deletion results in T cell lymphoproliferative autoimmunity which we have found includes increased T cell trafficking into the brain [18], by comparing PPI between the aforementioned subject groups, and standard C57BL/6-IL-2−/− (IL2-KO) and C57BL/6-IL-2+/+ (WT) mice, our experimental design enabled us to determine if the predicted alterations in PPI are due to loss of brain-derived IL-2, peripheral IL-2, or a combination of both factors.

## Materials and Methods

### Animals and congenic breeding

All mice in this study were cared for in compliance with the NIH Guide for the Care and Use of Laboratory Animals. Mice were housed in microisolater cages under specific pathogen free conditions. For GFP expression studies of the IL-2 transgene, female B6.Cg-Tg (IL2-EGFP) 17Evr (IL2p8-GFP) mice were obtained from the Mutant Mouse Regional Resource Center (University of Missouri) and bred in colony with C57BL/6 mice originally obtained from Jackson Laboratories. Transgene positive offspring were identified by PCR analysis of tail DNA. PCR primers in the IL-2 proximal promoter (IL2-1F: 5′-CATCCTTAGATGCAACCCTTCC-3′) and the GFP coding sequence (GFP-1R: 5′-GCTGAACTTGTGGCCGTTTAC-3′) were used, amplifying a 830-bp product in transgene-positive mice. PCR conditions were as follows: 95°C, 5 min, then 32 cycles of 95°C, 30 s; 58°C, 30 s; 72°C, 45 s, followed by a final 5 min at 72°C, using an i cycler (BioRad).

Congenic mice used for the behavioral studies were bred as described previously [19]. The IL-2 knockout mice (derived from 10 generations of backcrossing onto the C57BL/6 background) and C57BL/6-scid mutation mice were originally obtained from the Jackson Labs. Briefly, the breeding strategy was as follows. In the initial step, C57BL/6-IL-2+/− heterozygous and C57BL/6scid (homozygous for the SCID mutation) mice were crossed, resulting in mice heterozygous for both IL-2 and SCID, and then those mice were then backcrossed to SCID mice. Mice heterozygous for IL-2 and homozygous for the SCID mutation were then used as breeders to generate C57BL/6scid-IL-2−/− knockout (IL2-KO/SCID) and C57BL/6scid-IL-2+/+ (WT/SCID) littermates. C57BL/6-IL-2+/− × C57BL/6-IL-2+/− mice were crossed to generate IL2-KO (IL-2−/−) and WT (IL-2+/+) mice. The polymerase chain reaction (PCR) was used to genotype the offspring postweaning for IL-2 and immunoglobulin determinations were made to confirm the SCID mutation (Isostrip, Boehringer Mannheim) as described previously by our lab [19].

### Tissue preparation

Mice were anesthetized by intraperitoneal injection of a 0.5 mg/mL ketamine cocktail in a 3:3:1 ratio (ketamine/xylazine/acepromazine) and were perfused with 4% paraformaldehyde (PF). Brains were dissected, post-fixed in 4% PF for 2 hrs at room temperature, and cryoprotected in 30% sucrose overnight at 4°C. Tissue was snap frozen in isopentane and stored at −80°C. Coronal sections were cut throughout the brain and brainstem at a thickness of 30 μm. Sections were collected on Superfrost/Plus slides (Fisher Scientific) and stored at −80°C until staining could be performed.

### Immunohistochemistry

Tissue sections were air-dried and incubated in normal goat serum (Vector; 1:30 in PBS) for 1 hour at room temperature followed by overnight incubation at 4°C with the primary antibody rabbit anti-GFP (A-11122; 1:5000; Life Technologies). Phosphate buffer saline (1X) was used for all wash steps performed between incubation steps (Fisher Scientific). Visualization of the primary antibody was performed by incubating sections in goat anti-rabbit secondary antibody (1:2000, Vector Labs) for 2 hours at room temperature followed by incubation in avidin-peroxidase conjugates (1:500, Sigma) for 1 hour. The chromogen reaction was revealed by incubation in 3,3′-diaminobenzidine (DAB)-H_2_O_2_ solution (Sigma; 0.07% DAB/0.004% H_2_O_2_). Sections were counterstained with cresyl violet, dehydrated in ascending alcohol washes, cleared in xylenes, and cover slipped. Specificity of antibodies were tested by systematic omission of either primary or secondary antibody, no signal was obtained with each of the primary or secondary antibodies alone. Specificity of the anti-GFP primary antibody was further challenged by pre-incubating with recombinant GFP protein (23193; 5 ug/mL Rockland) prior to use in the staining protocol.

### Immunofluorescence

Tissue sections were air-dried and incubated in normal goat serum (Vector; 1:30 in PBS) for 1 hour at room temperature followed by overnight incubation at 4°C with the primary antibodies mouse anti- NeuN (MAB377; 1:250; Millipore), and rabbit anti-GFP (A-11122; 1:5000; Life Technologies). Phosphate buffer saline (1X) was used for all wash steps performed between incubation steps (Fisher Scientific). Visualization of the primary antibody was performed by incubating sections in goat anti-mouse Texas Red secondary antibody (1:300; Life Technologies), and goat anti-rabbit Alexa Fluor 488 secondary antibody (1:300; Life Technologies) for 2 hours at room temperature. Sections were coverslipped with Vectashield mounting medium (Vector Laboratories). All qualitative assessments of GFP staining in mice positive for the reporter were made in comparison to GFP negative littermates. The intensity of GFP expression was then ranked on a 4 level scale, where + was very low expression, ++ was low expression, +++ was medium expression, and ++++ was high expression.

### Acoustic startle reactivity and prepulse inhibition (PPI)

Two SR-LAB test chambers (San Diego Instruments) were used to measure acoustic startle response and prepulse inhibition as described previously [20]. Mice were placed in a small cylindrical enclosure (3.8×9.5 cm) located in a dark, ventilated chamber. A speaker located 30 cm above the cylinder delivered the background noise (65 dB), startle stimuli, and prepulse stimuli, all of which consisted of broadband white noise. Mice were allowed a 5-min acclimation period during which the background noise was delivered. Five different startle stimulus intensities (80, 90, 100, 110, and 120 dB) and two prepulse stimuli intensities (80 and 90 dB) paired with a 120-dB probe were presented in a pseudo-random sequence. Each was presented eight times for a total of 56 trials. The startle stimuli and the prepulse stimuli were of 30-ms duration and separated by 70 ms. Startle responses were recorded during a 100-ms period following the onset of each startle stimulus.

### Statistical analyses

Analysis of variance (ANOVA), and repeated measures ANOVA were used to make comparisions between subject groups. Post-hoc comparisons of interest were performed using the Fisher’s least significant difference test.

## Results

**Figure 1:**
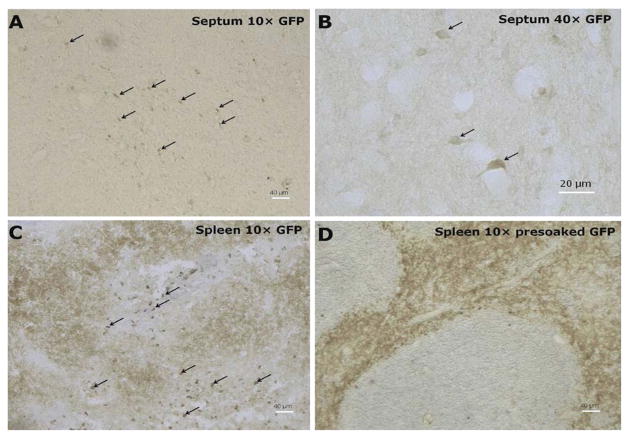
Photomicrographs of IHC stained GFP-positive cells in the septum (A and B; most GFP+ cells are in the lateral septum, with fewer in the medial septum), and the spleen (C) in IL2p8-GFP mice. Panel D illustrates that the reactivity of primary antibody was quenched in the spleen with 5 μg/ml free recombinant GFP protein prior to use in staining protocol.

**Figure 2:**
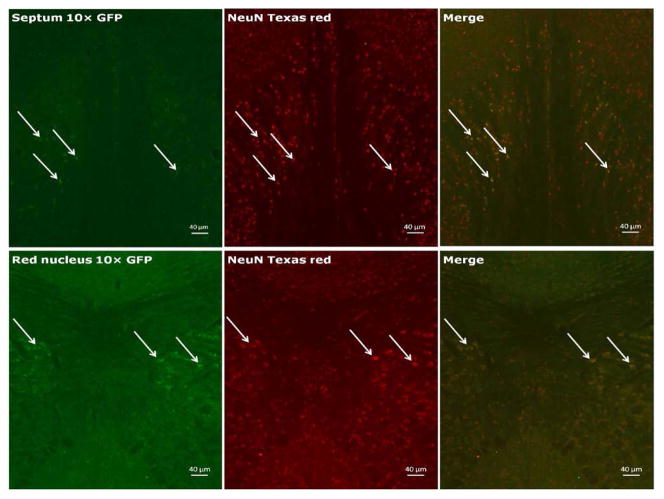
Representative photomicrographs showing expression of GFP+ cells in the septum (top: most GFP+ cells in the lateral septum and some in the medial septum) and red nucleus (bottom). Arrows label cells positive for GFP (left), the pan-neuronal cell marker NeuN (center), the co-localization of both markers (right).

**Figure 3:**
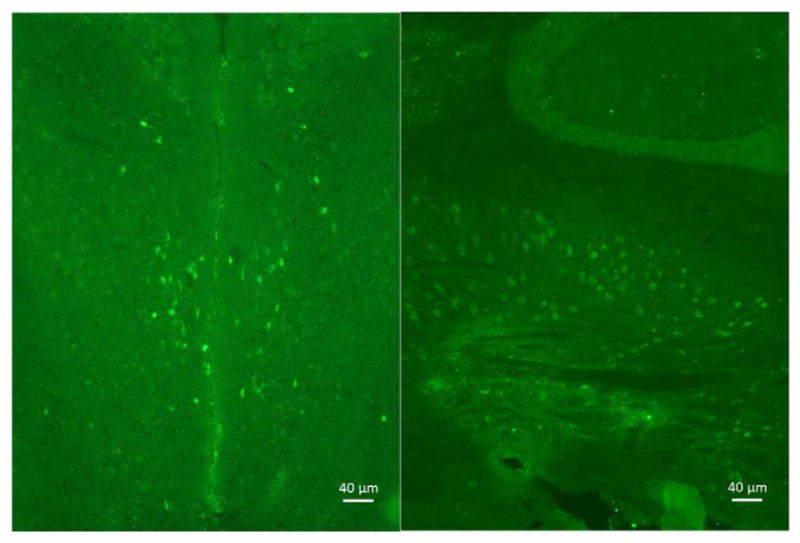
In the septum, most of the GFP-positive cells were found in the lateral septum, and some in the medial septum (left). GFP-positive cells were also found in fastigial nucleus and the interposed nucleus of the cerebellum (right). 10X magnification is shown here

We first tested the specificity of the GFP antibody by preincubating the primary antibody with recombinant GFP prior to use in our staining protocol to quench its ability to bind the antigen in tissue. As seen in Figure 1, as expected, immune cells (e.g., T cells, dendritic cells) in the white pulp area of the spleen expressed GFP, and the specificity of the primary antibody was clearly demonstrated by pre-incubation with recombinant GFP. As can be seen in Figure 2, a representative photograph of the septum and red nucleus, in most cells expressing GFP in the brain, the reporter was co-localized to the pan-neuronal cell marker, NeuN. Figure 3 shows GFP-positive cells identified in the medial and lateral septum, the fastigial nucleus, and the interposed nucleus of the cerebellum. In all areas examined, there were only a few cells that stained positive for GFP but not NeuN. Those brain cells were morphologically and geographically identical to brain cells expressing both markers. Across brain regions, we were able to determine relative expression of IL-2 in IL2p8-GFP transgenic mice. GFP expression was found throughout the rostral-caudal extent of the brain and brainstem of IL2p8-GFP transgenic mice in discrete nuclei, and with a wide range of staining intensity. The regional localization the IL2p8-GFP transgenic expression and its relative density in those regions are summarized in Table 1.

**Figure 4:**
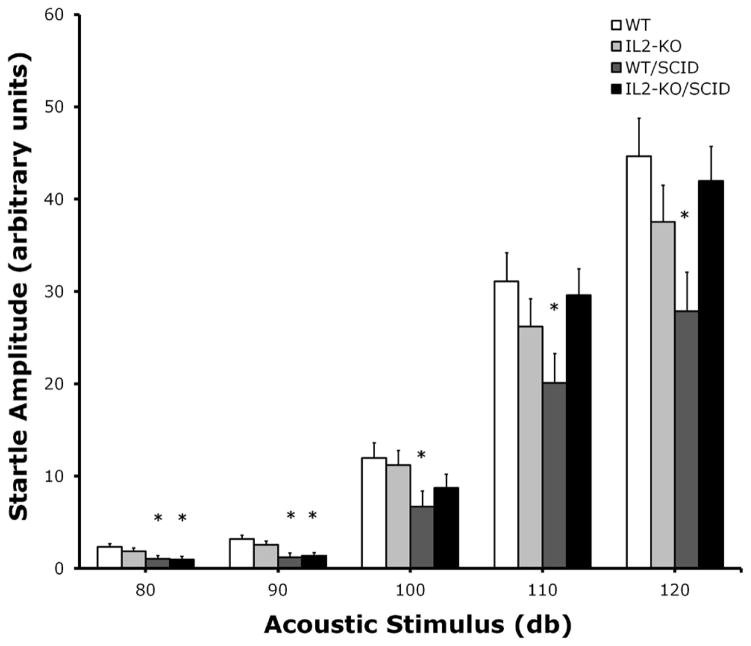
Comparison of acoustic startle reactivity in WT (n=23), IL2-KO (n=25), WT/SCID (n=22) and IL2 KO/SCID mice (n=28). Data are expressed as the mean ± S.E.M. of each subject group. *p < .05: at 80 db WT is different from WT/SCID and IL2-KO/SCID; at 90 db WT and IL2-KO are different than WT/SCID and IL2-KO/SCID; at 100 db WT is different than WT/SCID; at 110 and 120 db WT/SCID are different than WT and IL2-KO/SCID.

**Figure 5:**
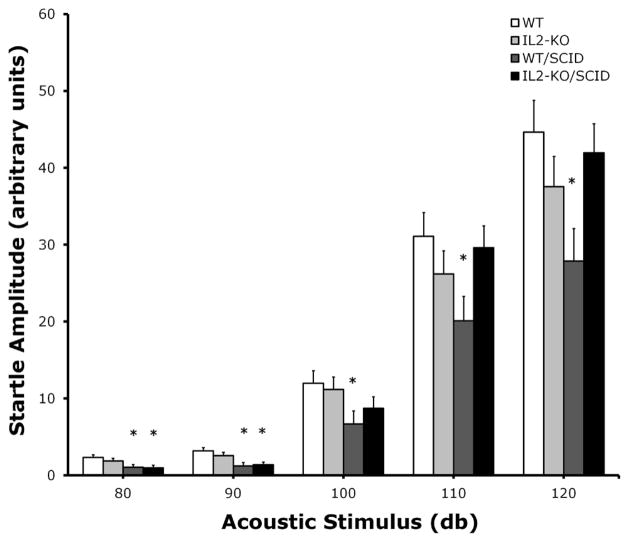
Comparison of prepulse inhibition (PPI) in WT (n=23), IL2-KO (n=25), WT/SCID (n=22) and IL2-KO/SCID mice (n=28). Data are expressed as the mean ± S.E.M. of each subject group. *p < .05, IL2-KO/SCID is different from all other subject groups.

Given the identification of IL2-GFP transgene in brain regions associated with regulation of sensorimotor gating including the striatum, lateral septum, the dorsal endopiriform nucleus, and the cingulate, we compared PPI between standard WT and IL2-KO, and the WT/SCID and IL2-KO/SCID congenic mice. Acoustic startle was assessed across five dB levels: 80, 90, 100, 110 and 120 dB. There was a significant main effect of subject group [F(3,94)=3.40, p<.05]. Overall, when looking at the data as depicted in Figure 4, across the different dB levels the WT/SCID mice had lower startle responses than the other subject groups. Post-hoc analyses was performed for each dB level and described in the figure legend. Figure 5 shows the results of the comparison of PPI across the groups, controlling for the effects of startle using mean startle amplitude as a covariate. At the 80 db prepulse level, there was a significant main effect of subject group [F(3,94)=3.07, p<.05]. Post-hoc analyses showed that the IL2-KO/SCID subject group was different from all of the other subject groups (*p<.05). At the 90 db prepulse level, there was not a significant main effect of subject group. Although the mean level of PPI was highest in the IL2-KO/SCID, none of the post-hoc comparisons between the subject groups at the 90 dB prepulse level were statistically significant.

## Discussion

These data indicate that IL2p8-GFP transgenic mice are a valuable model to determine the endogenous expression of IL-2 in the brain. The model had previously been shown to be a good tool to identify the expression of the cytokine in the peripheral immune system *in vivo* [15]. There are some reports suggesting that astrocytic and microglial enriched primary cultures in vitro may produce IL-2 [21,22], and of *in vivo* IL-2 expression from microglia in response to potent supraphysiologic stimuli such as lipopolysaccharide (LPS, a potent inducer of microglial activation) and hypoxic ischemia [23,24]. In the present study we evaluated the expression of GFP in the brains of IL2p8-GFP mice under normal physiological conditions, and did not detect GFP in any brain cell-types other than neurons. Future studies with this mouse model can determine if glial cell activation *in vivo* via LPS or hypoxic damage is required to induce IL-2 expression by endogenous brain glial cells, and clarify whether IL-2 expression by glia cell cultures may be due to the process of producing primary cultures from fetal brain tissue (and inconsistent with the physiology of the cytokine *in vivo*).

Multiple studies have shown that exogenously administered IL-2 to septohippocampal neurons has potent effects on the release of acetylcholine from septal neurons and trophic effects on both cell types in culture [2,3]. The distribution and enrichment of in situ IL-2 receptor gene expression in the septohippocampal system and related limbic regions corresponds with IL2-GFP transgene expression in IL2p8-GFP mice [11,12]. The lateral septum, where robust expression of GFP was detected, projects mainly to the medial septum and hippocampus and is therefore well positioned to provide modulatory input to the septohippocampal system. We also detected modest levels of GFP expression from a subset of cells in the subiculum, the main output structure of the hippocampus, that projects back to the septal nuclei (in addition to other GFP-positive regions including the prefrontal cortex, hypothalamus, mammillary nuclei, entorhinal cortex, and amygdala). Previously, we showed the effects of brain-derived IL-2 deficiency on cholinergic phenotype and neurotrophin expression in the septohippocampal system of IL-2KO mice [25,26]. In addition to the neuropathology in the septum and hippocampus, we have detected changes in several measures of behavior. Most notably, IL-2KO mice exhibited impaired spatial learning and memory in the Morris water maze [27]. Moreover, the entorhinal cortex is well established as the seat of spatial memory and navigation, whereas the dorsal endopiriform nucleus, lateral septum, amygdala, and cingulate are known to be involved in sensorimotor gating; all were found to express GFP in IL2p8-GFP transgenic mice. GFP was also expressed from the paraventricular hypothalamic nuclei that have direct projections to the pituitary gland, and the anterior/posterior hypothalamic nuclei involved in thermoregulation. These findings are in keeping with documented effects of exogenously administered IL-2 acting at different levels on the HPA axis [4,28–31]. Since IL-2 gene expression cannot be detected reliably in in different brain cell-types and regions using conventional in situ hybridization in the mouse, we were not able to confirm however that GFP-positive cells represent all the brain cells constitutively expressing endogenous IL-2.

Given the identification of IL2-GFP transgene in brain regions associated with regulation of sensorimotor gating including the lateral septum, dorsal endopiriform nucleus, entorhinal cortex, striatum, and the cingulate [16], we compared PPI between standard WT and IL2-KO, and the WT/SCID and IL2-KO/SCID congenic mice. We found that at the 80 db prepulse level, IL2-KO/SCID mice which are devoid of IL-2 gene expression in both the brain and the peripheral immune system, had increased levels of PPI compared to the other subject groups. We found previously that standard IL2-KO mice had increased PPI compared to WT mice. Here using these congenic IL2-KO/SCID mice, however, we were able to eliminate potential action of peripheral autoimmunity associated with IL-2 gene deletion. Though the directional change was the same (increased PPI) here and in our previous study using standard IL2-KO mice [27], in our first study we compared IL2-KO mice with WT colony controls supplied by Jax labs. By contrast, in the present study the background of the different subject groups was well controlled as all of the groups originated from the same initial breeding pairs. Although the effect of IL-2 on PPI remains to be elucidated further, particularly given the role of IL-2 on other factors that modify PPI such as dopamine release [32], together our studies suggests that loss of brain-derived IL-2 is associated with increased PPI, whereas loss of the common β-receptor gene for IL-2 and IL-15 results in decreased PPI [33]. In future research, it will be important to clarify further the role of IL-2 in sensorimotor gating, as well as examine other domains of behavior including learning and memory. We did not behaviorally test GFP expressing mice. Correlating IL2-GFP expression with different domains of behavioral performance in these transgenic mice could be a fruitful approach to relating changes IL2-GFP expression patterns to behavioral function.

Finally, it is notable that many nuclei that were found to express GFP are interconnected and have similar functional roles or modalities (Table 1). The motor cortex, anterior and lateral aspects of the striatum, interposed nuclei, red nuclei, inferior olivary nuclei, and the gigantocellular reticular nucleus, for example, are all involved in motor control. By contrast, the somatosensory cortex, fastigial nucleus of the cerebellum, vestibular nuclei, and the mesencephalic nucleus of the trigeminal nerve are important for proprioception. Lastly, many positive nuclei are involved in nociception and analgesia - periaqueductal gray, central gray of the pons, and the raphe magnus nucleus. Use of these transgenic mice in future research will help fill the gaps in our current knowledge of the origin of IL-2 in the brain, and elucidate further the role of brain-derived IL-2 in normal CNS function and disease.

## Figures and Tables

**Table 1:** List of nuclei positive for the IL-2 transgene throughout the rostral-caudal extent of the brain and brainstem. (+) symbol designates relative intensity of GFP staining as visualized by fluorescence immunohistochemistry.

Nucleus	Modality	Relative staining intensity
Mitral cell layer olfactory bulb	Olfactory	++
Granular cell layer olfactory bulb	Olfactory	+++
External piriform layer olfactory bulb	Olfactory	++
Anterior olfactory nucleus (ventral and medial)	Olfactory	++
Ventral and lateral orbital cortices	Limbic	++
Cingulate	Limbic	++++
Motor 1	Motor	++
Motor 2	Motor	++
Dorsal endopiriform nucleus	Sensory motor gating (SMG)	++++
Striatum	Motor, SMG	+
Lateral septum	Limbic, SMG	++++
Medial septum	Limbic	++
Horizontal limb diagonal band of Broca	Limbic	++
Subiculum	Limbic	+
Paraventricular hypothalamic nucleus	Limbic, Autonomic	+++
Basal lateral amygdaloid nucleus	Limbic	+
Anterior and posterior hypothalamus	Autonomic	++
Ventrolateral geniculate nucleus, parvocellular	Vision	+++
Nucleus of the solitary tract	Chemosensation	++++
Periaqueductal gray	Analgesia	++
Median raphe nucleus	Analgesia	++
Magnocellular reticular formation/ Gigantocellular reticular formation	Mixed	++++
Red nucleus	Motor	++++
Entorhinal cortex (medial/lateral)	Spatial memory, SMG	++++
Mammillary bodies	Memory	++++
Mesencephalic nucleus of 5	Proprioception, Motor	++
Pontine gray	Relay	++
Lateral vestibular nucleus	Proprioception, Motor	+++
Fastigial nucleus cerebellum	Proprioception, Motor	++++
Posterior interposed nucleus	Proprioception, Motor	++++
Inferior olivary nucleus	Proprioception, Motor	+++
